# Improving the Stability and Curcumin Retention Rate of Curcumin-Loaded Filled Hydrogel Prepared Using 4αGTase-Treated Rice Starch †

**DOI:** 10.3390/foods10010150

**Published:** 2021-01-13

**Authors:** Jihyun Kang, Ye-Hyun Kim, Soo-Jin Choi, Shin-Joung Rho, Yong-Ro Kim

**Affiliations:** 1 Department of Biosystems Engineering, Seoul National University, Seoul 08826, Korea; rkdwlgus0310@snu.ac.kr; 2 Division of Applied Food System, Major of Food Science & Technology, Seoul Women’s University, Seoul 01797, Korea; ye4978@swu.ac.kr (Y.-H.K.); sjchoi@swu.ac.kr (S.-J.C.); 3 Center for Food and Bioconvergence, Seoul National University, Seoul 08826, Korea; 4 Research Institute of Agriculture and Life Sciences, Seoul National University, Seoul 08826, Korea; 5 Global Smart Farm Convergence Major, Seoul National University, Seoul 08826, Korea

**Keywords:** 4αGTase-treated rice starch, curcumin, filled hydrogel, ultraviolet stability, curcumin retention

## Abstract

In this study, 4-α-glucanotransferase (4αGTase)-treated rice starch (GS) was added after 1-h (1 GS) and 96-h (96 GS) treatments to the aqueous phase of a curcumin-loaded emulsion to produce filled hydrogels (1 GS-FH and 96 GS-FH, respectively). The relative protective effects of the FH system, native rice starch-based filled hydrogel (RS-FH), and emulsion without starch (EM), on curcumin were evaluated based on ultraviolet (UV) stability and simulated gastrointestinal studies. The UV stability and curcumin retention after in vitro digestion of the filled hydrogels (FH) samples were greater than those of the EM samples. RS-FH showed a 2.28-fold improvement in UV stability over EM due to the higher viscosity of RS. 1 GS-FH and 96 GS-FH increased curcumin retention by 2.31- and 2.60-fold, respectively, and the microstructure of 96 GS-FH, determined using confocal laser microscopy, remained stable even after the stomach phase. These effects were attributed to the molecular structure of GS, with decreased amylopectin size and amylose content resulting from the enzyme treatment. The encapsulation of lipids within the GS hydrogel particles served to protect and deliver the curcumin component, suggesting that GS-FH can be applied to gel-type food products and improve the chemical stability of curcumin.

## 1. Introduction

Curcumin is a natural polyphenol compound extracted as a yellow pigment from the rhizome of *Curcuma longa* (turmeric), which has been used widely in food and many other industries because of its antioxidant, anti-inflammatory, and anticancer properties [1,2,3]. However, the applications of curcumin are severely limited by its low water solubility, bioavailability, and chemical stability [2,4,5,6,7,8]. Curcumin is hardly soluble in water at acidic or neutral pH, and undergoes rapid hydrolysis due to dissociation at alkaline conditions, resulting in a chemical instability [9,10,11]. Curcumin is also easily decomposed even in visible and ultraviolet light under storage conditions, producing many photolysis productions [5,12]. Moreover, curcumin has a very low percentage of ingested curcumin actually reaches to the blood due to its rapid degradation under physiological conditions [9,13,14].

Various systems, including emulsions, nanoemulsions, nanoparticles, liposomes, micelles, conjugates, molecular complexes, solid dispersions, and nanogels, have been studied to improve the solubility, stability and bioavailability of curcumin [15,16,17,18,19]. In particular, encapsulation within emulsion-based delivery systems, which are composed of oil droplets surrounded by an emulsifier dispersed within an aqueous phase, has been widely investigated to release curcumin at a desirable site of action or in accordance with a particular external stimulus, and to enhance its bioavailability [4,20,21,22,23]. They reported that trapping the curcumin within the oil phase of an emulsion-based delivery systems could help prevent it from degradation in the gastrointestinal tract (GIT) [20,22,23]. In addition, they suggested that the hydrolysates in the oil phase digested by enzymes in GIT formed mixed micelles along with bile acid and phospholipids, thereby increasing the solubility of curcumin transported to epithelial cells [4,21]. However, oil-in-water (O/W) emulsions are often susceptible to decomposition over time or when exposed to certain environmental stresses during production, storage, transport, and utilization [21]. Therefore, to overcome these problems, a new method of superimposing polysaccharides capable of increasing viscosity onto the aqueous phase or forming gel networks on O/W emulsions has been attempted recently [24]. This method is referred to as oil-in-water-in-water (O/W/W) emulsion, in which oil droplets are surrounded by hydrogel particles dispersed in an aqueous continuous phase containing a water-soluble emulsifier [21]. Polysaccharide-based hydrogels are physicochemical cross-linked macromolecules that form a three-dimensional network capable of holding water without disintegration and thus can serve as an ideal carrier for bioactive compounds [25,26].

**Figure 1:**
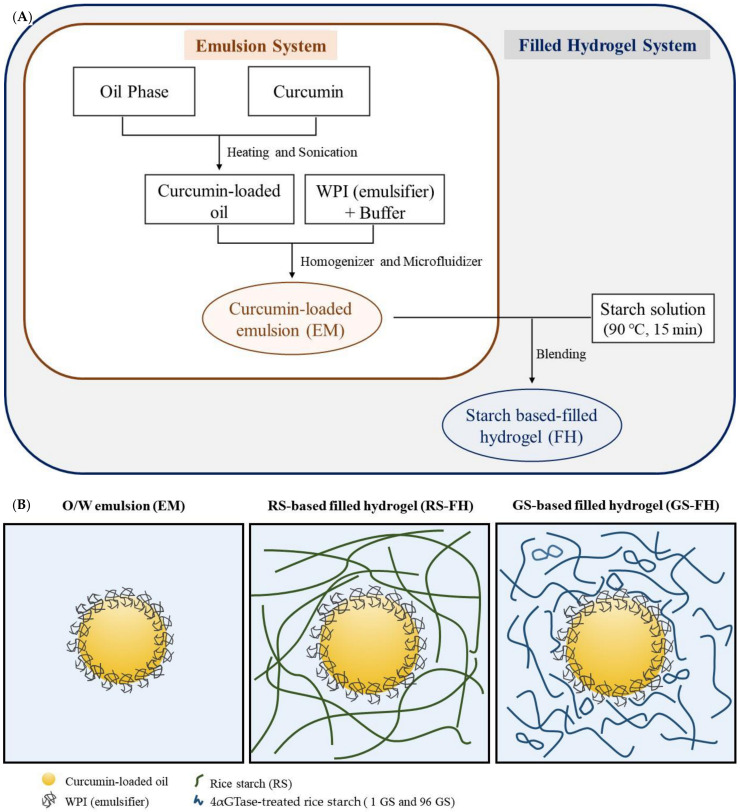
(**A**) Flow chart of manufacturing process for emulsion and filled hydrogel and (**B**) schematic representation of curcumin encapsulation systems. Left: oil in water (O/W) emulsion (EM), Middle: filled hydrogel prepared using rice starch (RS-FH), Right: filled hydrogel prepared using 4-α-glucanotransferase-treated rice starch (GS-FH).

Rice starch, as a natural polysaccharide capable of forming a hydrogel, can be used as a thickener, stabilizer, excipient, and fat mimetic in various foods [25,27]. Typically, normal rice starch forms a thermally irreversible gel with relatively high retrogradation rate as other cereal starches do, which limits its application as a gelling agent [25,28]. As an effort to overcome this limitation, many studies have attempted to change its physicochemical properties by enzyme treatment. In particular, it is known that enzymatic modification using 4-α-glucanotransferase (EC 2.4.1.25; 4αGTase), which can modify amylose and amylopectin molecules by cleaving and reforming α-1,4-glycosidic bonds, delays retrogradation of rice starch and improves stability [29,30,31]. The 4αGTase-treated rice starch showed improved freeze-thaw stability due to the reduction of the long chain amylose and modification of amylopectin side chain of native rice starch [30]. The unique structural modification of rice starch by enzyme could produce thermoreversible starch gel [30,32,33]. In a previous study, we used 4αGTase-treated rice starch (GS) that contained modified amylopectin clusters and cyclic glucans as a complexation agent to improve the solubility, stability, and bioaccessibility of hydrophobic compounds [34]. In addition, we have incorporated GS in the internal water phase of a water-in-oil-in-water (W/O/W) emulsion to improve heat and shear stability [35]. In this study, we attempted to use GS for filled hydrogel (FH) composed of oil droplets embedded in starch hydrogel in order to investigate the effect of its unique molecular structure on the encapsulation performance of FH. Therefore, the purpose of this study was to investigate the effects of GS-based filled hydrogel (GS-FH) on curcumin with respect to ultraviolet (UV) stability and curcumin retention and microstructural change after in vitro digestion. We prepared an emulsion (EM) without adding starch gel and a native rice starch-based filled hydrogel (RS-FH) for comparison. The production process and a schematic representation of the curcumin encapsulation system used in this study are detailed in Figure 1.

## 2. Materials and Methods

### 2.1. Materials

The rice starch was isolated from native rice (*Ilpumbyeo*) provided by the Rural Development Administration (Suwon, Korea) using a traditional alkaline extraction [36]. The 4-α-glucanotransferase (4αGTase) was produced as described in the previous study [37]. Curcumin (curcuminoid content ≥ 94%), mucin (from porcine stomach), α-amylase (from porcine pancreas), bile extracts (from porcine), pepsin (from porcine gastric mucosa), and pancreatin (from porcine pancreas, Type II) were purchased from Sigma-Aldrich (St. Louis, MO, USA). Soybean oil (Ottogi Corp., Pyeongtaek, Korea) was obtained from a local market. All other chemicals and reagents used were of analytical grade.

### 2.2. Preparation of 4αGTase-Treated Rice Starch

The 4αGTase-treated rice starch (GS) was prepared according to the previous study [34], with minor modification. Isolated rice starch was dispersed in distilled water (5%, *w*/*v*) and gelatinized at 95 °C for 30 min with mechanical stirring. The starch paste was then cooled to 75 °C mixed with 4αGTase (20 U/g, dry basis), and incubated at 75 °C for 1 h (1 GS) and 96 h (96 GS) with constant stirring. After the enzyme reaction was stopped by boiling for 10 min, the reaction solution was mixed with 5-fold volume of 95% ethanol. The precipitated starch was separated by centrifugation at 7167× *g* for 15 min and then dried in a dry oven at 40 °C.

### 2.3. Physicochemical Properties of 4αGTase-Treated Rice Starch

#### 2.3.1. Molecular Weight Distribution (HPSEC)

The molecular weight distributions of starch samples were determined by the high-performance size exclusion chromatography (HPSEC). Starch samples (RS, 1 GS, and 96 GS) dispersed in 90% dimethyl sulfoxide (DMSO) solution (1%, *w*/*v*) were boiled for 1 h and then stirred continuously for 24 h at room temperature. A 5-fold volume of 95% ethanol was added to the starch dispersions and centrifuged at 12,741× *g* for 15 min before drying the precipitate. The dried pretreatment samples were re-dissolved in boiling water (0.5%, *w*/*v*), filtered through a 5.0 μm disposable membrane filter, and then injected into a HPSEC system consisted of a refractive index detector (ProStar 355 RI Detector; Varian, Australia) and two running columns (OH-Pak 804 and OH-806 HQ; Shodex, Tokyo, Japan). Degassed distilled water (HPLC grade water, J.T.Baker, Deventer, Netherlands) was used as a mobile phase running at a flow rate of 0.4 mL/min. The molecular weight of the samples was assessed by comparing with those of the pullulan standard series (5900–788,000 Da, P-82, Showa Denko, Tokyo, Japan).

#### 2.3.2. Distribution of Branch Chain Length (HPAEC)

The pretreatment procedure of starch samples for high-performance anion exchange chromatography (HPAEC) analysis was exactly the same as for HPSEC analysis. The pretreated starch samples (0.25%, *w*/*v*) were incubated with 1 U/mg of isoamylase (Megazyme, Wicklow, Bray, Ireland) in 50 mM sodium acetate buffer (pH 4.5) at 40 °C for 2 h. The reaction solutions were filtered using a 0.45 μm membrane filter and analyzed using the HPAEC system with a CarboPac^TM^ PA1 column (4 × 250 mm, Dionex Corp., Sunnyvale, CA, USA). The filtered sample solutions were separated using gradient eluents with 0.15 M sodium hydroxide and 0.15 M sodium hydroxide in 0.6 M sodium acetate solution at a flow rate of 1.0 mL/min. The degree of polymerization (DP) of the samples was estimated using standard maltooligosaccharide mixture (Megazyme, Wicklow, Bray, Ireland) containing glucose (G1), maltose (G2), maltotriose (G3), maltotetraose (G4), maltopentaose (G5), maltohexaose (G6), and maltoheptaose (G7). The branch chain length distributions were calculated based on the relative peak area up to DP 63.

### 2.4. Encapsulation of the Curcumin in Filled Hydrogel

Curcumin-loaded O/W emulsion (EM) was manufactured by the procedure described in Ahmed et al. [3], with slight modification (Figure 1A). The oil phase was produced by adding curcumin in soybean oil (0.3%, *w*/*w*). For complete dissolution, curcumin contained oil was heated at 60 °C for 10 min and then sonicated (Ultrasonic Cleaner-Powersonic 410, Hwashin, Seoul, Korea) for 30 min [7,38]. A stock emulsion (30 wt% oil) was prepared by homogenizing curcumin-containing soybean oils and WPI solutions (WPI + 5 mM phosphate buffer (pH 7.0) (3%, *w*/*w*)) using a high-speed blender (ULTRA-TURRAX model T25 digital, IKA, Germany) for 2 min and then passing through a micro-fluidizer (Picomax MN 250 A, Micronox, Seongnam, Korea) three times at 10 kpsi. 

To prepare the filled hydrogels (FH), the starch sample was dispersed in distilled water (10%, *w*/*v*) and boiled at 90 °C for 15 min. Stock emulsion (30 wt% oil) was slowly poured into the hot starch solution while gently stirring using a glass rod to adjust the final oil concentration of the FH to 4%. The mixtures were stored at 4 °C overnight to form a gel.

### 2.5. Rheological Measurements

Rheological properties were evaluated according to Chen, Zhang, Li, Xie, and Chen [39] with some modifications. The dynamic viscoelastic properties of FH samples were conducted using an AR1500 ex rotational rheometer (TA Instruments Ltd., New Castle, DE, USA). The FH samples were transferred to the bottom plate and then pressed by upper parallel with a 1 mm gap between. After removing the excess sample, it was covered with a thin layer of low-density silicon oil to prevent evaporation losses during the experiment. The dynamic frequency sweep test was performed at a constant strain (0.5%), over a frequency range between 0.1 and 10 frequency (Hz) at room temperature. 

### 2.6. Texture Profile Analysis

Cylindrical gel samples (35 × 10 mm) were placed on the platform and a texture profile analysis (TPA) was carried out with a TA-HDi texture analyzer (Stable Micro Systems Ltd., Godalming, UK) using a P50 aluminum probe. The samples were compressed twice at a speed of 1 mm/s with a 40% strain. Samples in triplicates were tested for textural parameters to measure how the FH behaved when compressed to a large extent of deformation.

### 2.7. Ultraviolet Stability

Ultraviolet (UV) stability of curcumin encapsulated samples (EM, RS-FH, 1 GS-FH, and 96 GS-FH) was conducted under UV irradiation chamber. The samples were placed on a rotating plate and then exposed to UVB lamp (G8T5E, Sankyo-Denki, Kanagawa, Japan) emitting UV rays of 306 nm between 280 nm and 360 nm for up to 7 h. Subsequently, the FH samples were mixed with a 12-fold of acetone and centrifuged at 1210× *g* for 15 min. The EM samples used as a control were mixed with isooctane/2-propanol (3:2, *v*/*v*) and centrifuged at 1210× *g* for 3 min. The supernatant of the FH samples and bottom layer of EM sample were filtered through a 0.45 μm PVDF membrane filter, respectively, and analyzed using on a UV-vis spectrophotometer (UV-1650 PC, Shimadzu, Kyoto, Japan) at 430 nm. The curcumin content was estimated using a curcumin standard curve obtained for the corresponding solvent, and expressed as a curcumin retention (%), which was a percentage of the curcumin amount remaining in the sample after UV irradiation relative to the amount of curcumin in the sample before UV irradiation. All tests were performed in triplicate.

### 2.8. In vitro Digestion Test

A simulated gastrointestinal tract (GIT) model that consisting of oral, gastric, and intestinal phases was used to evaluate the biological fate of the encapsulated curcumin after in vitro digestion. This model was based on the method that has been described in detail in previous studies [34,40,41,42] with slight modifications. 

Oral phase: A solution for simulated saliva fluid (SSF) was prepared by mixing NaCl (159.4 mg), NH_4_NO_3_ (32.8 mg), KH_2_PO_4_ (63.6 mg), KCl (20.2 mg), K_3_C_6_H_5_O_7_ (30.8 mg), C_5_H_3_N_4_O_3_Na (2.1 mg), H_2_NCONH_2_ (19.8 mg), and C_3_H_5_O_3_Na (14.6 mg) in 100 mL of distilled water. A 15 mL of SSF solution was then mixed with 0.45 g of mucin and stirred overnight for a complete dissolution. Before starting the experiment, the solution was mixed with α-amylase (100 U/mL) warmed up to 37 °C. After adjusting the solution pH to 6.8 using 1 M NaOH solution, it is blended with each sample (curcumin, EM, and FH) at a 1:1 ratio and incubated at 37 °C for 10 min with constant stirring. In the case of FH, soft gel samples were crushed sufficiently using a spatula in the mixture to mimic mastication.

Gastric phase: Simulated gastric fluid (SGF) was prepared by dissolving 2 g of NaCl, and 7 mL of HCl (37%) in 1 L of distilled water and then adding 3.2 g of pepsin. After 15 mL aliquot of SGF was mixed with 15 mL of the oral phase digesta, the pH was adjusted to 2.5 using 1 M HCl. The mixture was then incubated at 37 °C for 2 h with continuous stirring. 

Small intestinal phase: After completion of the gastric digestion phase, 30 mL of the stomach digesta were adjusted to pH 7 and 3.5 mL of bile extract solution (187.5 mg/3.5 mL) dissolved in 50 mM phosphate buffer (pH 7) and 1.5 mL of salt stock solution (10 mM of CaCl_2_ and 150 mM of NaCl) were then added. The mixed solution was adjusted once again to pH 7 and 2.5 mL of pancreatin solution (187.5 mg/2.5 mL) dissolved in 50 mM phosphate buffer (pH 7) was added, followed by incubation at 37 °C for 2 h with stirring constantly. The change in pH of the mixed solution was monitored and the volume of 0.25 M NaOH titrated to maintain the pH at 7 during the 2 h incubation was recorded, using a pH-stat automatic titration unit (Orion 420 A+, Thermo Electron Corp., Beverly, MA, USA) [43].

### 2.9. Measurement of Curcumin Retention Rate and Released Free Fatty Acids in the In Vitro Digestion System

Curcumin retention was quantified as the remaining amount of curcumin in digesta after in vitro digestion system. The raw digesta, collected after the small intestinal stage, were then mixed with chloroform at 1:1 ratio and centrifuged at 315× *g* for 10 min using a centrifuge (Supra 22 K, Hanil Science Inc., Incheon, Korea). The extracted curcumin was collected from the bottom layer, and examined on a UV-vis spectrophotometer at 430 nm with pure chloroform as a blank. The initial samples before digestive process were determined as a control. The curcumin retention rate was calculated in the following Equation (1):

(1) $$\mathrm{Curcumin}\;\mathrm{retention}\;\mathrm{rate}\;\left(\%\right)=\frac{\text{Curcumin concentration in raw digesta}}{\text{Initial curcumin concentration}}\times 100$$

Free fatty acid (FFA) released due to fat digestion were measured by the volume of NaOH solution required to neutralize the sample, and expressed as a percentage using the following Equation (2):

(2) $$\%\;\mathrm{FFA}=100\times \frac{V_{\mathrm{NaOH}}\times m_{\mathrm{NaOH}}\times M_{\mathrm{lipid}}}{w_{\mathrm{lipid}}\times 2}$$

where *V*_NaOH_ is the volume (L) of NaOH, *m*_NaOH_ is the molarity of NaOH (M), *M*_lipid_ is the molecular weight of soybean oil (920 g/mol), and *w*_lipid_ is the oil weight (g) in the digestion system.

### 2.10. Confocal Laser Scanning Microscopy

Structural changes of oil droplets during the digestive process were measured using confocal laser scanning microscopy (Leica TCS SP8 X, Leica Micro-systems, Wetzlar, Germany). The samples acquired from the different stages (initial, mouth, stomach, and intestine) of the GIT were dyed with Nile red dissolved at 0.01% (*w*/*v*) in ethanol. The fluorescently labelled samples were placed on glass slide and examined using a 63 × oil immersion objective lens. Nile red was excited at a wavelength of 488 nm. All captured images were processed using the Leica software program (Leica application suite X, LAS X, Leica, Wetzlar, Germenay).

### 2.11. Statistical Analysis

All the experiments were performed duplicate or triplicate and presented as the mean and standard deviation (mean ± SD). All data were analyzed by the one-way ANOVA test followed by a Duncan’s multiple range test using SPSS for windows (ver. 25.0, IBM Corp., Armonk, NY, USA). A value of *p* < 0.05 was considered statistically significant between the data. 

## 3. Results and Discussion

### 3.1. Molecular Structural Properties of 4αGTase-Treated Rice Starches 

The molecular weight (M_w_) distributions of RS and 4αGTase-treated rice starches (1 GS and 96 GS, respectively) are shown in Supplementary Materials Figure S1. The M_w_ distribution of RS had a bimodal shape: fraction I (retention time, 18–32 min) with high M_w_ in accordance with amylopectin and fraction II (retention time, 32–45 min) with lower M_w_ corresponding to amylose [27]. Fraction I of GS decreased gradually and the fraction II region of GS increased with longer enzyme treatment time, which is consistent with the results of Do et al. [27]. After 96 h of enzyme treatment (96 GS), the highest peak M_w_ of fraction II had shifted from 2.12 × 10^5^ g/mol to 2.28 × 10^4^ g/mol. This indicated that GS was composed of polymers that were hundreds to thousands of times smaller than that of the native starch. This result was attributed to the production of amylopectin clusters with reduced size as a result of rearrangement of the amylopectin inner and outer branch chains of the rice starch via the disproportionation action of the 4αGTase [44]. In addition, previous research reported that the amylose content of rice starch was significantly reduced by 4αGTase. Therefore, amylopectin as well as amylose molecules could contribute to the decrease in relative M_w_ by hydrolysis [33,37].

The branch chain length distributions of RS, 1 GS, and 96 GS measured by high-performance anion exchange chromatography (HPAEC) are presented in Supplementary Materials Figure S2. The chain length distributions of RS before 4αGTase treatment represented a sharp horn-like shape with degree of polymerization (DP) 12 at the apex, whereas those of GS showed a more gradual and flat distribution from DP 3 to DP 62. The most conspicuous alteration caused by the enzyme was that the relative proportions of A chains, B_2_ chains, and B₃ chains increased, whereas those of B_1_ chains decreased, which is consistent with a previous study [45]. 4αGTase rearranges parts of the amylose and amylopectin molecules by cleaving and reforming α-1,4- and α-1,6-glycoside bonds [45]. In other words, this enzyme acts on the external branch chains (A and B_1_ chains) of amylopectin, causing the chain length to be redistributed more evenly, and modifies the internal chains (B_2_ and B_3_ chains) of amylopectin, promoting their involvement in the interconnection of short chain clusters [27]. Thus, the increase in long-branch chains (B_2_ and B_3_ chains) may be due to the cleaved amylose and short-branch chains of amylopectin (B_1_ chains) being shuffled to other non-reducing ends of amylopectin chains via catalysis of intermolecular transglycosylation by the enzyme [46]. In addition, the slight increase in the proportion of A chains (DP ≤ 12) after enzyme treatment was probably due to the partial hydrolysis and/or the formation of cyclic glucans by intramolecular rearrangement of branch chains of amylopectin [45]. Meanwhile, the relative portion of the long-branch chains (B_2_ and B_3_ chains) of 96 GS was reduced slightly (1 GS, 31.15%; 96 GS, 29.66%) and that of the short-branch chains was increased (1 GS, 31.19%; 96 GS, 34.00%) compared to 1 GS. This could be due to the cleavage of the internal chains and/or the formation of cyclic glucans by intramolecular transglycosylation and also the trimming of the external chains by the increased enzyme treatment time [45,47].

### 3.2. Rheological Properties of Filled Hydrogel

The dynamic rheological properties of the FH samples (RS-FH, 1 GS-FH, and 96 GS-FH) were analyzed using rheometer to measure the storage (G′) and loss (G″) modulus, which are the main parameters useful for characterizing the viscoelastic properties of semi-solid hydrogels. G′ is a measure of a mechanical energy stored that can be recovered per deformation cycle, representing samples’ elastic property. G″ refers to an estimate of the energy dissipated as heat per deformation cycle, indicating viscous property of the sample. The tan δ is the value obtained by dividing G″ by G′. The higher tan δ indicates more liquid-like and less solid-like behavior, and vice versa [48,49,50].

**Figure 2:**
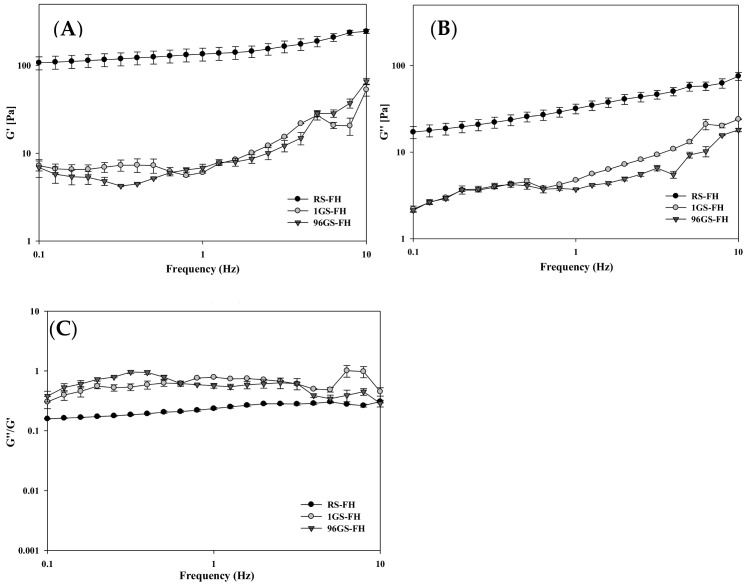
Rheological properties of filled hydrogel samples [native rice starch-based filled hydrogel (RS-FH), 4-α-glucanotransferase treated (for 1 h) rice starch-based filled hydrogel (1 GS-FH), and 4-α-glucanotransferase treated (for 96 h) rice starch-based filled hydrogel (96 GS-FH)] during a frequency sweep (0.1–10 Hz) at 25 °C. The dynamic shear properties of the filled hydrogel were measured by (**A**) storage modulus (G′), (**B**) loss modulus (G″), and (**C**) loss tangent (tan δ = G″/G′). The error bars represent the mean and standard deviations of triplicate experiments.

G′ values for all samples were higher than G″ values over all measured frequency range, indicating that the sample is primarily solid-like (Figure 2). However, dynamic rheological parameters of RS-FH and GS-FH were significantly different. Considerably high moduli (G′ and G″) values as well as tan δ (G″/G′) values greatly lower than 1 for RS-FH indicated strong gel formation. In contrast, GS-FH showed fairy low moduli values and tan δ (G″/G′) values close to 1, which indicated weak gel formation [51]. G′ was related to the association degree of the gel network, and G′ almost the same or slightly higher than G″ was a typical feature of weak gel [52]. This significant difference in gel properties between RS-FH and GS-FH was attributed to the molecular structure modification of the GS samples by enzyme treatment. Typical starch hydrogels have a three-dimensional network due to the association of amylose and amylopectin molecules, showing some degree of elasticity [53]. In enzyme-treated starch hydrogels, the gel network changed due to molecular structural modification [37,54]. As mentioned in Section 3.1, 4αGTase essentially degraded most of amylose molecules and decreased the molecular weight of amylopectin via inter- and intramolecular transglycosylation. As a result, the degree of association decreased in GS-FH, and a weak gel formed.

### 3.3. Textural Properties of Filled Hydrogel

We performed texture profile analysis (TPA) to compare the textural properties of the FH samples. The mean values of the textural parameters are shown in Table 1. The hardness differed significantly among FH samples and was markedly lower for 96 GS-FH. The gumminess (hardness × cohesiveness) and chewiness (hardness × cohesiveness × springiness), which are mainly affected by hardness, also showed statistically low results for 96 GS-FH. These results might be attributed to changes in the amylose and amylopectin structure of GS caused by the enzyme treatment. As described for the HPSEC and HPAEC results, the amylose molecules of 1 GS and 96 GS were hydrolyzed, and the ratio of amylopectin with a longer branch chain length distribution (DP > 25) increased due to the enzyme reaction (Supplementary Materials Figures S1 and S2). In particular, the average DP value of the 96 GS amylose region (approx. DP 130) was also significantly lower than that of RS (approx. DP 980–1110) [55]. A previous study reported that the high hardness of RS was related to high amylose content, leading to crystallization in a short period of time [56]. The amylose content in GS decreased markedly, and the proportion of small amylose and even smaller molecules increased greatly. Therefore, 96 GS formed a weak gel due to a significantly low molecular weight, which contributed to a relatively low hardness. Other parameters, adhesiveness and elasticity, did not differ significantly among FH samples.

### 3.4. Ultraviolet Stability of the Curcumin Encapsulation System

**Figure 3:**
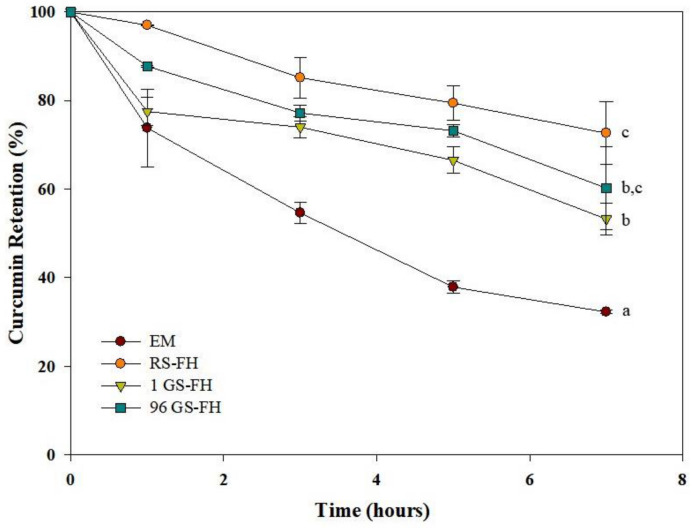
Photo-stability of curcumin encapsulated with emulsion (EM), native rice starch-based filled hydrogel (RS-FH), 4-α-glucanotransferase treated (for 1 h) rice starch-based filled hydrogel (1 GS-FH), and 4-α-glucanotransferase treated (for 96 h) rice starch-based filled hydrogel (96 GS-FH) during ultraviolet B (UVB) exposure for 7 h. The curcumin retention (%) is a percentage of the curcumin amount remaining in the sample after ultraviolet irradiation relative to the initial curcumin amount before UV irradiation. The error bars represent the mean and standard deviations of triplicate experiments. Results marked with the different letter are significantly different (*p* < 0.05).

Curcumin is very unstable and degraded quickly under ultraviolet (UV) irradiation due to its structure with two hydroxy methoxyphenyl rings linked by two β-diketone groups [5,34,57]. Therefore, in this study, we prepared FHs using GS (1 GS and 96 GS) to improve the stability of curcumin. As shown in Figure 3, the amount of curcumin remaining in all samples decreased gradually as the UV irradiation time was extended. FH was more stable than EM against UV irradiation at all times. After 7 h of UV irradiation, approximately 60% of curcumin in EM was destroyed, whereas more than 50% of curcumin was retained in FH. When exposed to UV radiation in both solution and the solid state, curcumin mainly loses two hydrogen atoms, forming a cyclization product of curcumin [2]. Degraded curcumin acts as a photosensitizer of singlet oxygen and undergoes self-sensitized decomposition. A previous study reported that several radicals induced by UV irradiation in an emulsion system may oxidize or react with charged groups and thus neutralize the charge distributed on the interface of oil droplets of the emulsion, thereby deteriorating stability [38]. In addition, the study also revealed that coating the emulsion with polymers could more effectively protect curcumin from oxidation and degradation. Therefore, the FH used in this study might also protect curcumin by providing a physical barrier that blocks the passage of UV rays [38].

Among the FH samples, RS-FH showed the highest protective effect, with higher curcumin retention (>72.6%) after 7 h of UVB exposure. This may be due to the fact that RS has a faster gelation rate at room temperature, leading to the formation of a harder gel network than GS and thereby increasing the UV blocking effect, even though gel opacity of RS-FH and GS-FH was hard to be visually differentiated (Supplementary Materials Figure S3). GS-FH (1 GS > 53.3%; 96 GS > 60.3%) also showed a significant curcumin protection effect against UV. GS was initially maintained in a liquid state during preparation; however, with time, the solution acquired the unique rheological properties that form a gel [30,37]. Therefore, this result might be due to GS forming a gel network in the O/W emulsion, which prevents the release of the curcumin present therein. Previous studies have reported the possibility of using emulsifiers in emulsions of starch and/or enzymatically modified starch as gelling polymers [35]. They found that when enzymatically modified starch was included in the W/O/W internal water phase, the encapsulation efficiency and stability of the emulsion could be improved while reducing the average diameter of the oil phase. 

Meanwhile, 96 GS-FH (approx. 60.3%) had a significantly higher curcumin protective effect than 1 GS-FH (approx. 53.3%). In the molecular weight distribution diagram of GS (Supplementary Materials Figure S1), 96 GS consisted of amylopectin clusters and had a more uniform molecular weight distribution than 1 GS. Therefore, 96 GS gel formed a well-organized and dense physical barrier, thereby increasing the protective effect for curcumin, as in our previous study [34]. Our results suggested that GS-FH, as well as RS-FH, can be used as a potential well material to improve the stability of curcumin under UV, thereby extending its shelf-life during storage.

### 3.5. Lipid Digestibility of the Curcumin Encapsulation System

**Figure 4:**
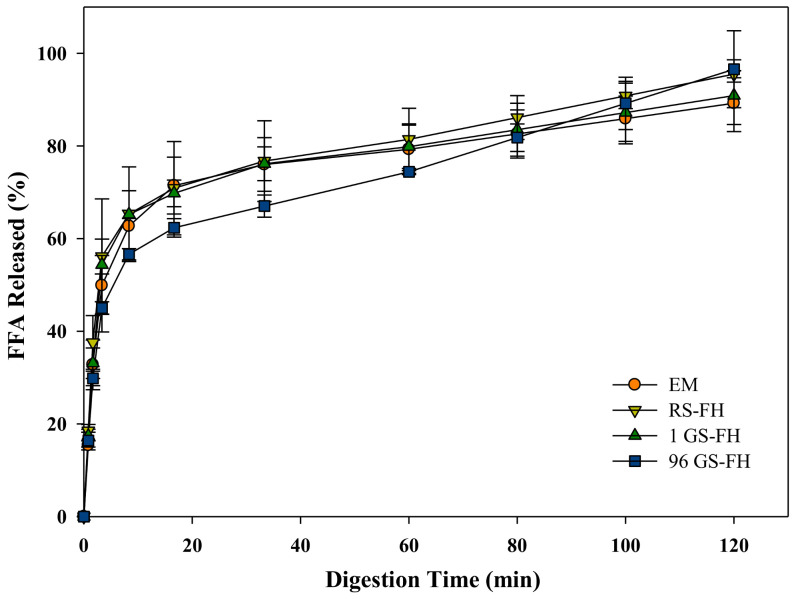
Release profiles of free fatty acid (FFA) released (%) from curcumin-loaded in emulsion (EM), native rice starch-based filled hydrogel (RS-FH), 4-α-glucanotransferase treated (for 1 h) rice starch-based filled hydrogel (1 GS-FH), and 4-α-glucanotransferase treated (for 96 h) rice starch-based filled hydrogel (96 GS-FH). The error bars represent the mean and standard deviations of triplicate experiments.

The amount of free fatty acid (FFA) released during intestinal digestion after gastric condition was measured to compare emulsion and filled hydrogel systems (Figure 4). The lower the FFA released (%), the less the lipid degradation by lipase, indicating slower digestion process. The FFA released in all samples increased rapidly during the first 10 min, followed by a more progressive increase at longer times (Figure 4). The amounts of FFAs released at 120 min ranged from 89% to 97%. These results indicated that FFAs were being generated in these samples as a consequence of the conversion of triacylglycerols into two free fatty acids and monoacylglycerols by the action of lipase [58]. The final extents of lipid digestion after 60 min were fairly similar among all samples. The digestion rate of 96 GS was not statistically significant, but was somewhat slower during the initial 60 min, as shown by the results presented in the graph (Figure 4). This result implied that the lipase in the aqueous phase was difficult to access the surface of the encapsulated lipid, probably because 96 GS with modified molecular structure more densely covered the oil droplets in FH [59]. An increase in transit time in the gastrointestinal tract due to the slower rate of lipid digestion may prolong the period of release and absorption of bioactive substances in the small intestine [60,61,62]. However, the rate and extent of lipid digestion among samples did not differ significantly because the 4% of oil content in emulsion and FH might not be enough to affect the digestibility results.

### 3.6. Curcumin Retention Rate in the Curcumin Encapsulation System after In Vitro Digestion

**Figure 5:**
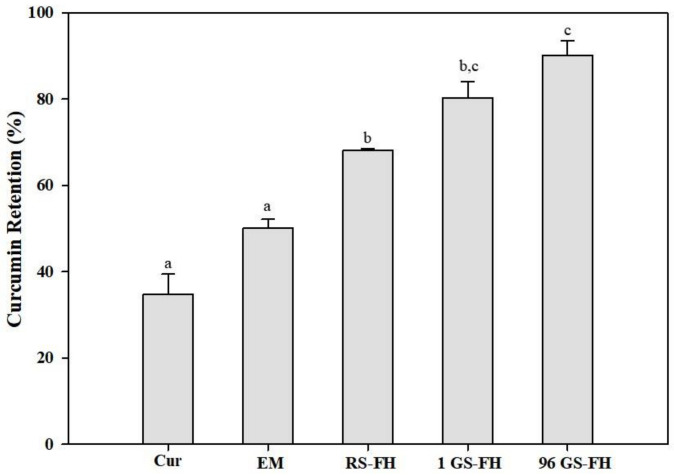
Retention rate (%) of curcumin (Cur), and curcumin-loaded in emulsion (EM), native rice starch-based filled hydrogel (RS-FH), 4-α-glucanotransferase treated (for 1 h) rice starch-based filled hydrogel (1 GS-FH), and 4-α-glucanotransferase treated (for 96 h) rice starch-based filled hydrogel (96 GS-FH) after in vitro digestion. The error bars represent the mean and standard deviations of triplicate experiments. Results marked with the different letter above the bars are significantly different (*p* < 0.05).

The influence of the presence or absence of the starch particles in emulsion on curcumin retention under simulated physiological environmental conditions was determined (Figure 5). The curcumin retention (%) refers to the amount of compound that actually remains in the food matrix during passage through the gastrointestinal tract. During the gastrointestinal digestion, a synergistic interaction between enzyme and pH conditions resulted in significant breakdown of curcumin (retention rate: approx. 34.7%). The degradation of curcumin is caused by partial deprotonation in aqueous solution and this reaction is known to be caused by direct contact with the aqueous phase [63]. The curcumin encapsulation system clearly had a significant effect on curcumin retention after the digestive process (*p* < 0.05). Generally, in the absence of lipid digestion products, the absorption rate of the hydrophobic bioactive compounds after digestion is relatively low because the bile salts and phospholipids in the intestinal fluids only form simple mixed micelles in the intestinal fluid [4,64]. Because the curcumin encapsulation systems prepared in our study contained lipids to form mixed micelles in the intestine, a significant comparison of curcumin retention was possible. 

As shown in Figure 5, the EM and RS-FH systems retained at least 50.1% and 68.1% of the encapsulated curcumin, respectively, following digestion, showing a slower and more efficient release than the control (free curcumin). The EM result may be attributed to increased formation of mixed micelles, in which curcumin can be dissolved and transported under simulated small intestine conditions, as demonstrated in the previous study [65]. Both the 1 GS-FH (approx. 80.2%) and 96 GS-FH (approx. 90.1%) systems showed significant curcumin protection due to the effect of the hydrogel, which prevented droplet aggregation in the gastrointestinal tract, as shown in previous studies by confocal laser microscopy [66]. In particular, the 96 GS-FH droplets were more effectively wrapped by a small molecular weight polymer, which could increase the stability of the emulsion and prevent the decomposition of curcumin within the digestive model system, especially in the stomach phase of strong acidity. Based on these results, it can be expected that 96 GS-FH prevented curcumin degradation by protecting the emulsion under the strong acidic condition and inhibiting pepsin from degrading the emulsifier (WPI) in the stomach phase through the reversible aggregation characteristic of 96 GS. RS and GS used in FH differed significantly in molecular weight distribution and gelation properties (Section 3.1 and Section 3.2). GS showed a higher curcumin protective effect than RS, but the precise underlying molecular mechanisms require further research. The FH system maintained a semi-solid structure even after exposure to digestion because of the gel network, which increased curcumin stability and the formation of mixed micelles. It is thought that this high level of retention due to the formation of mixed micelles will significantly increase the bioaccessibility of curcumin.

### 3.7. Changes in the Microstructure of Curcumin Encapsulation Systems during In Vitro Digestion

**Figure 6:**
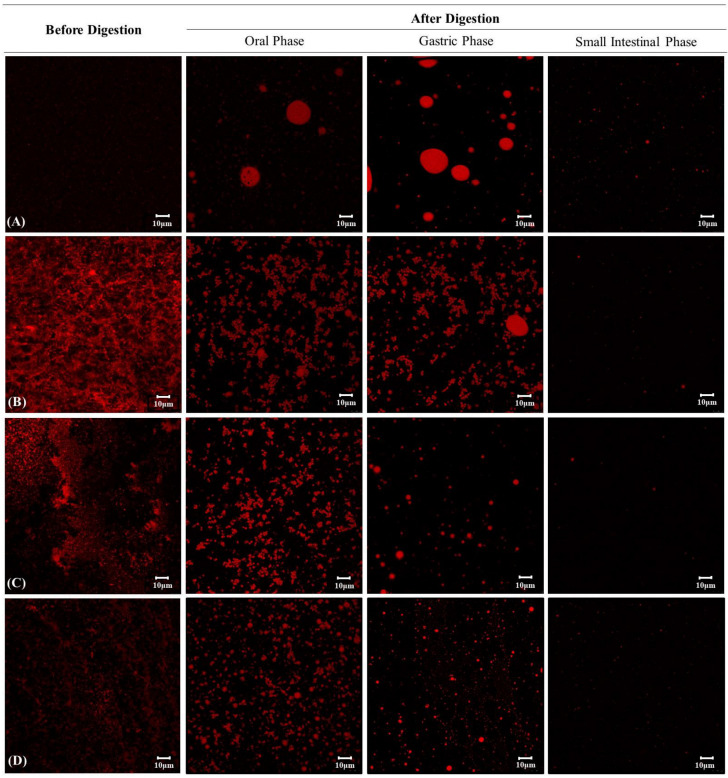
Microstructural changes in (**A**) emulsion (EM), (**B**) native rice starch-based filled hydrogel (RS-FH), (**C**) 4-α-glucanotransferase treated (for 1 h) rice starch-based filled hydrogel (1 GS-FH), and (**D**) 4-α-glucanotransferase treated (for 96 h) rice starch-based filled hydrogel (96 GS-FH) determined using confocal microscopy during in vitro digestion. Photographic images indicate the appearance of the sample before digestion and after digestion at the stages of the oral, stomach and small intestine phases. The red parts represent the lipid and the scale bar represents the length of 10 μm.

Finally, we assessed the microstructural changes of oil droplets in the curcumin encapsulation systems with confocal laser scanning microscopy during in vitro digestion system (Figure 6). In the EM system, the oil droplet size increased, as some coalescence of oil droplets was produced in the oral and gastric phases, and the confocal image disappeared in the small intestine phase due to oil droplet digestion by lipase (Figure 6A). Some coalescence and flocculation of ingested oil droplets in the oral and gastric phases may be attributed to the depletion flocculation and bridging mechanism caused by non-accreted mucin in the simulated saliva [59,67]. These mucin molecules produced an osmotic attraction between the oil droplets, which allowed them to come together, resulting in a change in particle size [68].

Meanwhile, FH samples exhibited some flocculation of the oil droplets in the oral phase, but they showed differences in microstructure in the gastric phase depending on the hydrogel type (Figure 6B–D). The fine oil droplets of RS-FH showed both coalescence and flocculation at the oral phase but were dispersed quite uniformly throughout. However, substantial coalescence of oil droplets was observed upon exposure to gastric juice (Figure 6B). As illustrated in Figure 6C,D, GS-FH showed only slight coalescence, not only in the gastric phase but also in the oral phase, suggesting that the oil droplets were still trapped within the hydrogel network. As mentioned above (Section 3.1), GS had weaker gel strength than RS, but it could form a compactly packed gel network with a relatively reduced molecular weight and narrow molecular weight distribution. In addition, the gel network structures of RS-FH and GS-FH differ. RS-FH forms a distinct three-dimensional network structure, whereas GS-FH produces a weak gel condition in which GS molecules are evenly distributed around the oil due to the quite low molecular weight of GS. Therefore, GS may have filled the emulsion oil more densely, slowing the diffusion of reactants, catalysts, and products involved in the lipolysis process. A previous study reported that starch-based hydrogel prevented the aggregation of oil droplets by forming a highly viscous aqueous phase that delayed droplet movement [66]. Our results also showed that partial hydrolysis of the starch gel network by the action of α-amylase in the oral phase affected the oil protective effect in the samples. In addition, the starch particles prevented the hydrolysis of WPI by pepsin in the gastric phase, so fewer large-size oil droplets formed by coalescence were observed than in the EM system. In this study, we confirmed that the unique gelation properties of GS acted as an important factor influencing the stability of the oil.

## 4. Conclusions

In this study, we confirmed that 4αGTase-treated rice starch-based filled hydrogel (GS-FH) significantly improved the curcumin retention (*p* < 0.05). The UV stability of curcumin with FH was improved as much as 2.28-fold compared to that of the EM system. In addition, 1 GS-FH (80.2%) and 96 GS-FH (90.1%) improved the stability and curcumin retention rate in the in vitro digestion system compared to free curcumin (34.7%) and EM (50.1%) samples. The improved curcumin retention with GS may be attributed to an overall increase in the number of amylopectin long branch chains and a reduction in molecular weight, which was related to a unique gel structure with effective protection of curcumin containing oil droplets in FH. Therefore, GS could successfully protect curcumin dissolved in the oil phase of the emulsion in its hydrogel form under various environmental conditions with pH variation. 

Overall, FH system made of GS, a rice starch structurally modified by 4αGTase, can provide useful tools when designing effective delivery systems for various hydrophobic bioactives such as curcumin in food and pharmaceutical industries. The fact that 4αGTase-treated starch is considered as a clean label starch currently on the market [46,69] can provide an additional benefit for its applications. Possible applications of FH made of GS include a biocompatible carrier for oral administration of sensitive functional food ingredients [70]. Also, the application can be extended to functional food coatings and packaging materials to enhance the shelf-life of food products [71]. Lastly, even though the improvement of curcumin stability in GS-FH under simulated gastrointestinal conditions and an environmental light stress, which was evidenced in this study, can be meaningful as it is, in order to evaluate its final efficacy as a delivery system, in vivo bioavailability test is the necessary next step.

## Figures and Tables

**Table 1:** Textural properties of filled hydrogel (RS-FH, 1 GS-FH, and 96 GS-FH).

FH Samples ^1^	Gel Characteristics					
	Hardness [N]	Adhesiveness	Cohesiveness	Springiness (%)	Gumminess [N]	Chewiness [N]
RS-FH	5.17 ± 0.10 ^c2^	1.56 ± 0.07 ^NS3^	0.68 ± 0.02 ^b^	93.53 ± 0.27 ^NS^	3.52 ± 0.17 ^c^	329.45± 14.91 ^c^
1 GS-FH	4.10 ± 0.05 ^b^	1.80 ± 0.28	0.35 ± 0.01 ^a^	94.66 ± 11.34	1.45 ± 0.08 ^b^	136.90 ± 9.21 ^b^
96 GS-FH	1.18 ± 0.13 ^a^	1.75 ± 0.20	0.43 ± 0.04 ^a^	86.91 ± 2.47	0.50 ± 0.00 ^a^	43.56 ± 1.06 ^a^

All values were expressed as means of triplicate determinations ± SD. ^1^ RS-FH, curcumin-loaded emulsion to native rice starch-based filled hydrogel; 1 GS-FH and 96 GS-FH, curcumin-loaded emulsion to 4-α-glucanotransferase-treated (for 1 h and 96 h, respectively) rice starch-based filled hydrogels. ^2^ Results marked with the different letter (a, b, c) in a column are significantly different (*p* < 0.05). ^3^ NS: no significant difference.

## Data Availability

The data presented in this study are available on request from the corresponding author.

## Supplementary Materials

The following are available online at https://www.mdpi.com/2304-8158/10/1/150/s1, Figure S1: Molecular weight distributions of native rice starch (RS) and 4-α-glucanotransferase-treated rice starch (1 GS and 96 GS). The molecular weight (Da) of the pullulan standard was shown in the upper right. Figure S2: Relative branch chain length distribution of (**A**) native rice starch (RS), (**B**) rice starch treated with 4-α-glucanotransferase for 1 h (1 GS), and (**C**) rice starch treated with 4-α-glucanotransferase for 96 h (96 GS). A chain: DP ≤ 12, B_1_ chain: DP 13-24, B_2_ chain: DP 25-36, B_3_ chain: DP > 36. Figure S3: Visual appearance of the prepared curcumin loaded in native rice starch-based filled hydrogel (RS-FH), 4-α-glucanotransferase treated (for 1 h) rice starch-based filled hydrogel (1 GS-FH), and 4-α-glucanotransferase treated (for 96 h) rice starch-based filled hydrogel (96 GS-FH).
