# Supplementary material for: Improving the Stability and Curcumin Retention Rate of Curcumin-Loaded Filled Hydrogel Prepared Using 4αGTase-Treated Rice Starch
Source: Foods. 2021 Jan 13;10(1):150. doi: 10.3390/foods10010150 (PMC7828239; doi:10.3390/foods10010150)
Supplement: Supplementary file 1 [file foods-10-00150-s001.pdf]

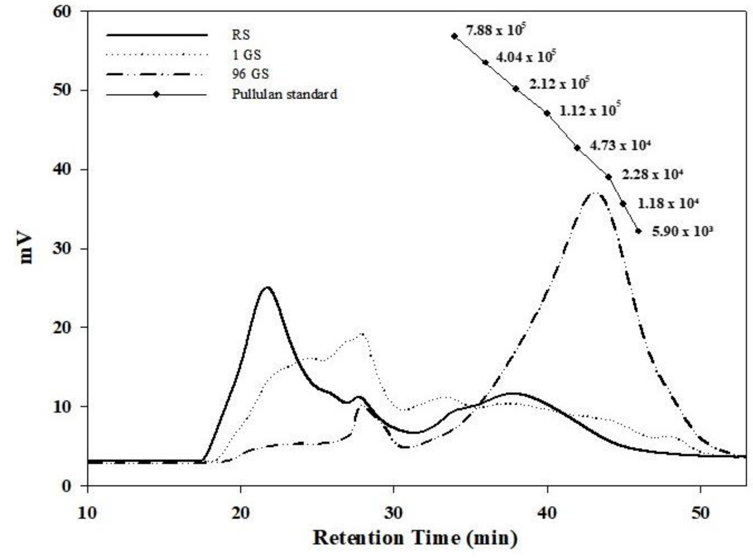

Figure S1. Molecular weight distributions of native rice starch (RS) and 4-α-glucanotransferase-treated rice starch (1 GS and 96 GS). The molecular weight (Da) of the pullulan standard was shown in the upper right.

(A)

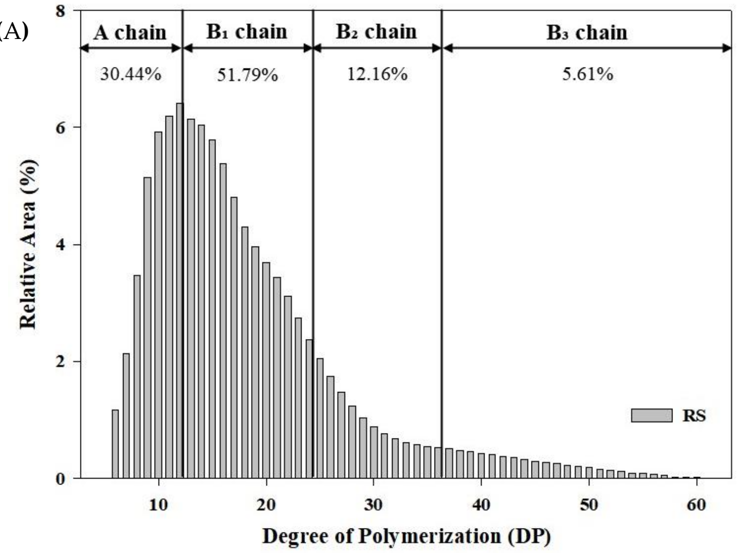

(B)

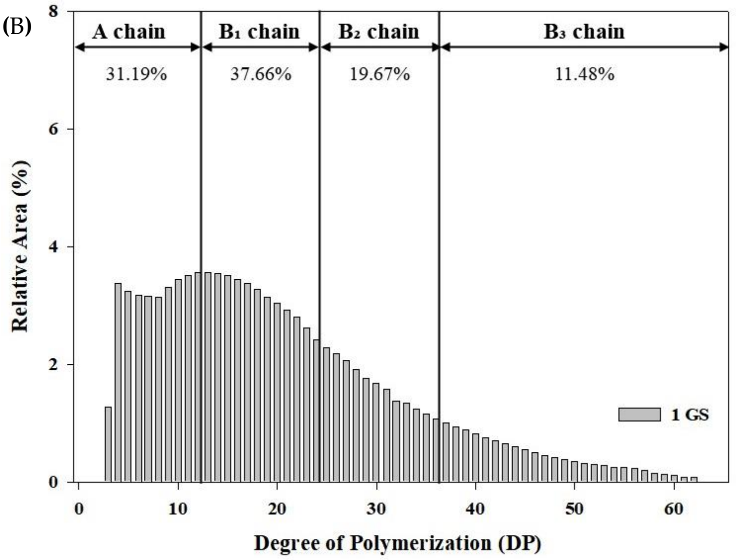

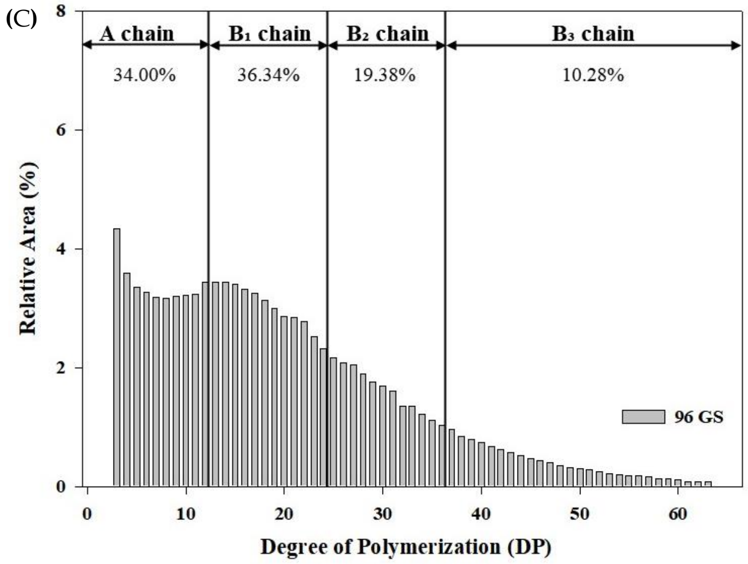

Figure S2. Relative branch chain length distribution of (**A**) native rice starch (RS), (**B**) rice starch treated with 4-α-glucanotransferase for 1 h (1 GS), and (**C**) rice starch treated with 4-α-glucanotransferase for 96 h (96 GS). A chain: DP ≤ 12, B_1 chain: DP 13-24, B_2 chain: DP 25-36, B_3 chain: DP > 36.

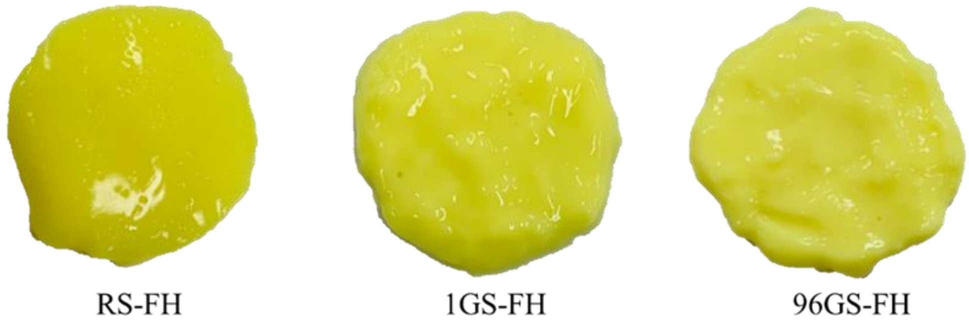

Figure S3. Visual appearance of the prepared curcumin loaded in native rice starch-based filled hydrogel (RS-FH), 4-α-glucanotransferase treated (for 1 h) rice starch-based filled hydrogel (1 GS-FH), and 4-α-glucanotransferase treated (for 96 h) rice starch-based filled hydrogel (96 GS-FH).
